# Strengthening the Merci Mon Héros Campaign Through Adaptive Management: Application of Social Listening Methodology

**DOI:** 10.2196/35663

**Published:** 2022-06-28

**Authors:** Martha Silva, Jonathan Walker, Erin Portillo, Leanne Dougherty

**Affiliations:** 1 Department of International Health and Sustainable Development School of Public Health and Tropical Medicine Tulane University New Orleans, LA United States; 2 Fluency M&C Saatchi New York, NY United States; 3 Johns Hopkins Center for Communication Programs Baltimore, MD United States; 4 Population Council Washington, DC United States

**Keywords:** social media, health communication, young people, reproductive health

## Abstract

**Background:**

Between 2014 and 2018, the penetration of smartphones in sub-Saharan Africa increased from 10% to 30%, enabling increased access to the internet, Facebook, Twitter, Pinterest, and YouTube. These platforms engage users in multidirectional communication and provide public health programs with the tools to inform and engage diverse audiences on a range of public health issues, as well as monitor opinions and behaviors on health topics.

**Objective:**

This paper details the process used by the U.S. Agency for International Development–funded Breakthrough RESEARCH to apply social media monitoring and social listening techniques in Burkina Faso, Côte d’Ivoire, Niger, and Togo for the adaptive management of the Merci Mon Héros campaign. We documented how these approaches were applied and how the lessons learned can be used to support future public health communication campaigns.

**Methods:**

The process involved 6 steps: (1) ensure there is a sufficient volume of topic-specific web-based conversation in the target countries; (2) develop measures to monitor the campaign’s social media strategy; (3) identify search terms to assess campaign and related conversations; (4) quantitatively assess campaign audience demographics, campaign reach, and engagement through social media monitoring; (5) qualitatively assess audience attitudes, opinions, and behaviors and understand conversation context through social media listening; and (6) adapt campaign content and approach based on the analysis of social media data.

**Results:**

We analyzed posts across social media platforms from November 2019 to October 2020 based on identified key search terms related to family planning, reproductive health, menstruation, sexual activity, and gender. Based on the quantitative and qualitative assessments in steps 4 and 5, there were several adaptive shifts in the campaign’s content and approach, of which the following 3 shifts are highlighted. (1) Social media monitoring identified that the Facebook campaign fans were primarily male, which prompted the campaign to target calls to action to the male audience already following the campaign and shift marketing approaches to increase the proportion of female followers. (2) Shorter videos had a higher chance of being viewed in their entirety. In response to this, the campaign shortened video lengths and created screenshot teasers to promote videos. (3) The most negative sentiment related to the campaign videos was associated with beliefs against premarital sex. In response to this finding, the campaign included videos and Facebook Live sessions with religious leaders who promoted talking openly with young people to support intergenerational discussion about reproductive health.

**Conclusions:**

Prior to launching health campaigns, programs should test the most relevant social media platforms and their limitations. Inherent biases to internet and social media access are important challenges, and ethical considerations around data privacy must continue to guide the advances in this technology’s use for research. However, social listening and social media monitoring can be powerful monitoring and evaluation tools that can be used to aid the adaptive management of health campaigns that engage populations who have a digital presence.

## Introduction

In 2020, there were an estimated 3.8 billion social media users worldwide and approximately 5.2 billion smartphone users [1]. Between 2014 and 2018, the penetration of smartphones in sub-Saharan Africa increased from 10% to 30%, enabling increased access to the internet [2]. Although internet penetration is lower in low- and middle-income countries (LMIC) than high-income countries, those in LMIC who have access to the internet through any devices are more likely to network using social media platforms [3]. Facebook, Twitter, Pinterest, and YouTube are the dominant social media platforms in most of francophone West Africa [4]. These platforms engage users in multidirectional communication and provide public health programs with the tools to inform and engage diverse audiences on a wide range of public health issues, as well as monitor opinions and behaviors on health topics [5,6]. Public health campaigns routinely include social media advertisements, create fan pages, and promote conversations on social media around campaign topics [7]. However, it is only in the last decade that evidence on the use of social media for health behavioral change campaigns has emerged in the literature for LMIC [8], with the focus primarily on reducing tobacco use [9], supporting patients undergoing HIV treatment [10], influencing sexual health behaviors [11], and influencing behaviors related to infectious diseases such as malaria [12]. Among the few studies focusing on adolescent sexual and reproductive health (SRH) behaviors, most address the acceptability of using social media to interact with young people and do not assess the extent to which social media campaigns have reached their intended audience and influenced health behaviors [13,14]. Studies using social listening techniques in LMIC have only recently emerged due to the relevance of these tools to monitor the COVID-19–related infodemic [15].

Adolescent pregnancy remains a major contributor to maternal and child mortality and intergenerational cycles of ill-health and poverty [16]. West and Central Africa have the highest annual adolescent birth rate in the world at 129 live births per 1000 young women aged 15-19 years, and the lowest use of modern contraception among all women at 16% [17,18]. Merci Mon Héros (MMH), or “Thank You My Hero” in French, is a multimedia campaign codeveloped and implemented by youth activists and the U.S. Agency for International Development (USAID)–funded West Africa Breakthrough ACTION (WABA) projects. WABA is a regional, USAID-funded initiative aiming to increase the coordination and effectiveness of social and behavioral change interventions in 4 priority countries: Burkina Faso, Côte d’Ivoire, Niger, and Togo. The MMH campaign is designed for youth and adults, with the aim of promoting an environment conducive to young people’s informed, voluntary family planning (FP) and reproductive health (RH) service access in francophone West Africa. The youth-led campaign videos (described in Table 1) and other content such as quizzes, concerts, and recorded conversations are disseminated via social media platforms, including Facebook, Instagram, Twitter, and YouTube, as well as through more traditional channels, such as television, radio, community activities, and others.

The USAID-funded Breakthrough RESEARCH project, in partnership with M&C Saatchi, collaborated with WABA to apply social listening and social media monitoring as part of a multimethod adaptive management and impact evaluation strategy of the MMH campaign. Social media monitoring refers to quantitatively tracking mentions and comments on social media regarding a specific topic, whereas social listening allows public health campaigns to better understand the context of web-based interactions by qualitatively tracking and analyzing conversation content [19].

This paper details the process of applying social media monitoring and social listening for the adaptive management of the MMH campaign in 4 countries: Burkina Faso, Côte d’Ivoire, Niger, and Togo. We documented how social media monitoring and social listening were applied to inform the MMH campaign and how the lessons learned can be used to support future public health campaigns.

**Table 1:** A sample of Merci Mon Héros campaign videos and Facebook Live events analyzed.

Video	Primary message
Gracian	Talk about sexuality with young people, without shame, from an early age so they can engage in healthy sexual and reproductive health behaviors
Camara	Prepare young women for puberty and menarche with accurate information Talk to young people about sexual and reproductive health
Florence	Young people need to know how to protect themselves Use a condom during sex to avoid the risk of sexually transmitted infections such as HIV
Mariette	Girls need to be informed and educated about menstruation so they can prepare themselves psychologically and manage periods effectively
Serge	Talk with youth about life goals and priorities so they can make reproductive health and family planning choices accordingly
Fanta	Provide accurate information to young people about sexual and reproductive health and the onset of menstruation
Oury	Inform youth about family planning options to avoid unintended pregnancy, and support rather than shame youth in the event of an unplanned pregnancy
Kouamé	Encourage young people to visit a health provider to learn more about contraceptive methods and choosing one that is right for them
Sedjro	Partner communication about family planning is important, and family planning decision-making should be shared
Mme Camara	Contraceptive methods can help plan pregnancies Select or switch family planning methods as needed according to your current needs and priorities
Aichatou	Parents should speak openly with young people about sexual and reproductive health, regardless of the parent’s or child’s sex or gender
Facebook Live events	Female puberty Male puberty The menstrual cycle and calculating the fertile window

## Methods

### Applying Social Listening to the MMH Campaign

The process of applying social media monitoring and social listening to the MMH campaign involved 6 steps: (1) ensure there is a sufficient volume of topic-specific web-based conversation in your target countries; (2) develop measures to monitor the campaign social media strategy; (3) identify search terms to assess campaign and related conversations; (4) quantitatively assess campaign audience demographics, campaign reach, and engagement through social media monitoring; (5) qualitatively assess audience attitudes, opinions, and behaviors and understand conversation context through social media listening; and (6) adapt campaign content and approach based on the analysis of social media data. Data requirements, procedures, considerations, and illustrative results are described under each step of the outlined process.

### Ethics Approval

This study obtained exempted status from the Population Council Institutional Review Board (EX2019011).

### Step 1: Ensure There Is a Sufficient Volume of Topic-Specific Web-Based Conversation in Your Target Countries

When starting any social listening exercise, it is necessary to first establish whether there is a sufficient volume of conversation to analyze. Typically, this is done by conducting a quick exploratory search of web-based content using a select group of keywords. This search string can be enhanced at a later point (see step 3)—at this point the purpose is simply to ensure that conversation does exist. There is no expected benchmark for the volume of posts, as this will vary substantially based on topic and review period. Broadening search terms if a limited volume of conversation is found may be useful for exploratory purposes. However, as the search string is honed and rules are defined, the sample of relevant posts will be reduced. If social listening is used to assess changes in web-based conversation before and after an intervention, nonexistent or limited content can still serve as a baseline measure.

### Step 2: Develop Measures to Monitor the Campaign Social Media Strategy

The MMH campaign was designed to create “surround sound” coverage through multiple channels and reach its 2 priority audiences—young people aged <24 years and adults aged ≥25 years who support and interact with them—in different ways. The goal of sharing content through social media channels was to leverage this space to normalize the habit of talking about youth SRH and contraception needs and empower young people to share their own stories and seek the information that would help them make informed, voluntary FP choices for their future. A team of young campaign designers from francophone Africa provided input into the channel selection. Facebook, Instagram, and YouTube were selected because they were considered the social media platforms most used by young people in the region. Throughout the campaign, Facebook was the most consistently used platform by MMH audiences, and as such, the web-based campaign strategy was largely developed with Facebook’s format in mind (ie, short videos, quizzes, static images, frequency of posts, and livestreams). Twitter was included in the web-based strategy to reach relevant organizations and decision-makers. To contribute to MMH’s “brand” on the internet and with an aim of being enveloped into existing web-based SRH conversations, the campaign created 2 hashtags—#MerciMonHéros and #BrisezLesTabous (“break taboos” in French)—and complementary topical (eg, hashtag #sexualité) and video-specific (eg, hashtag #Héros2Mariette) hashtags for each of the first 5 campaign videos.

We identified conversation volume as a key indicator to track changes over the time for topic-specific social media posts and comments related to FP, RH, and other relevant subtopics such as puberty and menstruation. Key indicators were also selected to help us track social media users’ interaction with the campaign. Table 2 defines the 4 indicators identified at the start of the campaign to assess progress: conversation volume, reach, engagement, and views.

**Table 2:** Social media indicators.

Indicator	Definition
Conversation volume	The number of social media posts pertaining to a specific topic (ie, menstruation, etc).
Reach	The number of screens that viewed the MMH^a^ videos.
Engagement	The number of times people engaged with MMH posts through reactions, comments, shares, retweets, mentions, and likes. Engagement can occur through paid promotion or when social media users organically find the campaign content.
Views	The number of MMH video views of at least 30 seconds, where each video is at least 2 minutes long.

^a^MMH: Merci Mon Héros.

### Step 3: Identify Search Terms to Assess Campaign and Related Conversations

To analyze social media content thematically, we defined search terms to identify social media conversations related to the campaign’s topics of interest. The Breakthrough RESEARCH team developed a set of relevant keywords to capture conversations about behaviors supporting young people’s conversations about and access to FP and RH services. The keywords included but were not limited to first sex, condoms, contraception, menstruation, and pregnancy, etc. These keywords were then shared with local youth stakeholders through Breakthrough ACTION to ensure we captured not only the correct usage in the local French language but also any known slang versions. These translated and context-specific search terms were entered into a Boolean search string—a type of search that allows users to combine or exclude keywords—designed to identify conversations across social media that were most relevant to the selected search terms. We used Crimson Hexagon’s BrightView algorithm for text analysis software to analyze social media data [20]. The Crimson Hexagon software searched all public-facing social media for relevant conversations, including mentions from the Facebook campaign page, Twitter, social newsfeeds, blogs, forums, Reddit, Tumblr, and YouTube. Privacy limitations relating to Facebook and Instagram only allow a very limited number of posts to be included in the analysis beyond the Facebook campaign page. We collected social media content from October 2018 to October 2019 (baseline) and from November 2019 to January 2021 (initial campaign implementation period). Given that certain keywords generate a sizeable volume of irrelevant conversation, we used 2 techniques to minimize irrelevant conversation. By filtering our search and tying the keywords of interest to pronouns (eg, “I,” “my,” “his,” and “her,” etc), a substantial volume of irrelevant noise was cleaned from the results, ensuring the sample contained posts more suitable for qualitative analysis. In addition, we used machine learning technology to train the software to reduce irrelevant content by training our algorithm to classify a selection of social posts into key topic areas. Once a representative sample was completed by human classification, the machine learning algorithm then analyzed the remaining untrained posts and classified them accordingly based on the language and content detected in the posts.

### Step 4: Quantitatively Assess Campaign Audience Demographics, Campaign Reach, and Engagement Through Social Media Monitoring

We used social media monitoring techniques to quantify campaign engagement and track conversation volume during campaign implementation. Using demographic characteristics associated with user profiles, we further disaggregated campaign platform engagement by age, sex, and geographic location to understand the audience’s demographic characteristics.

### Step 5: Qualitatively Assess Audience Attitudes, Opinions, and Behaviors and Understand Conversation Context Through Social Media Listening

We used Brandwatch, a social listening tool that enables analysts to investigate the data in various ways and at a granular level using topic wheels, word clouds, topic clustering, and bigram analysis. Data visualization options within the tool allow users to identify emerging themes. Some of the different techniques we used are outlined below:

- Topic wheel: This allows analysts to view the most frequently recurring keywords and phrases, which helps to easily identify how the main research themes relate to subthemes.
- Word clouds: Word clouds enable analysts to identify the most important and newly trending words, hashtags, emojis, and associated entities (people, places, and organizations) in the query.
- Topic clustering: Topic clustering displays topics and subtopics for segments of the overall data. The topics visualized in the output are selected based on how unique they are to the chosen segments. Clusters can be further filtered to identify positive or negative sentiment.
- Bigram analysis: A bigram analysis uses unstructured text data and measures how often words occur next to each other in text. This is a useful tool to identify emerging themes for further qualitative exploration.

Social listening findings validated the relevance of prioritized campaign topics (ie, the importance of encouraging honest dialogue about menstruation between parents and youth). We also quantified topic-specific conversation volumes for comparison at baseline (from October 2018 to October 2019) and endline (from November 2019 to January 2021) to assess if topic-specific conversation was increasing.

### Step 6: Adapt Campaign Content and Approach Based on the Analysis of Social Media Data

After using data visualization techniques to analyze the general social media conversation and campaign-specific engagement, we shared reports with WABA to inform evidence-based adaptations to the MMH campaign.

## Results

**Figure 1:**
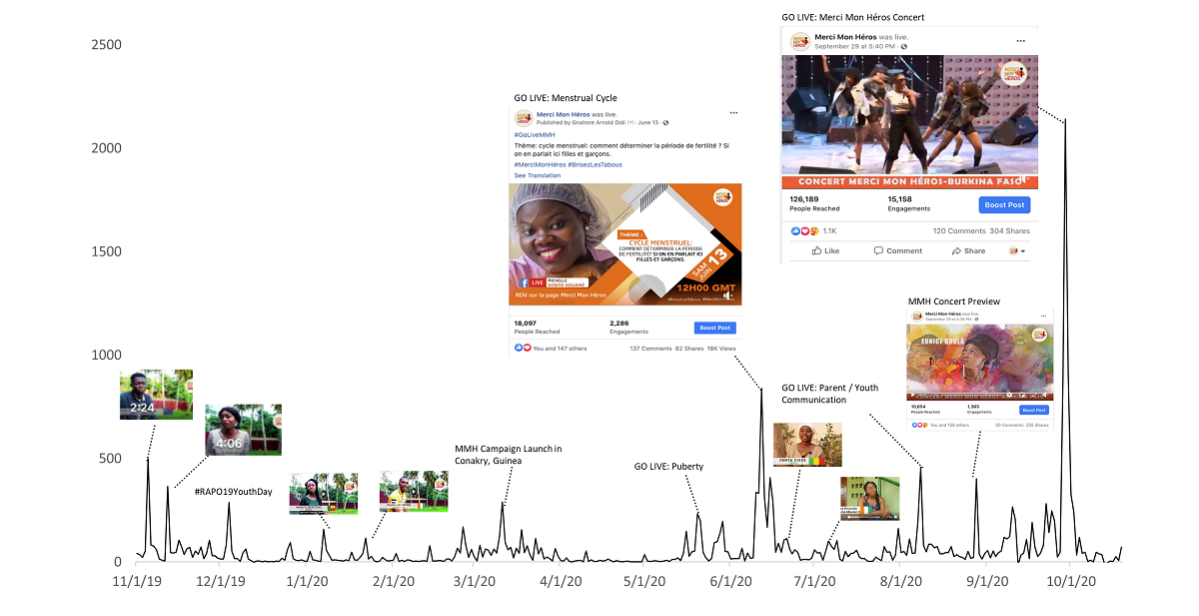
Merci Mon Héros conversation volume over the implementation period (from November 1, 2019, to October 20, 2020).

Figure 1 summarizes the conversation volume over the course of implementation and highlights when spikes in conversations related to campaign video themes occurred on social media. We found that 71% (20,611/29,030) of campaign followers were male social media users, with 60% (17,418/29,030) of the total users aged <24 years.

Table 3 shows the geographic distribution of campaign engagement by Facebook users, which, interestingly, does not mirror the levels of internet penetration. Among all Facebook users who engaged with the campaign content, most (32.28%, 937/2903) are from Ouagadougou, followed by Lomé (16.12%, 468/2903), Abidjan (14.16%, 411/2903), and Niamey (4.79%, 139/2903). Routine monitoring of Facebook page views indicated that although paid promotion of the campaign content garnered more campaign reach, organic viewers had better campaign video completion rates than paid promotion viewers (viewers who watched campaign videos to the end: 3.9%, 1031/26,435 vs 0.7%, 185/26,435, respectively).

The reports we shared with WABA provided extensive information related to trends in the conversation volume, campaign engagement, relevant hashtags, and extensive anonymized content data with direct quotes from user-generated content (see Table 4). Findings from the reports were used for program refinement throughout the initial MMH campaign period and beyond the social media monitoring time frames (beyond January 2021).

**Table 3:** Distribution of Facebook users who engaged with the Merci Mon Héros campaign content by city.

City	Users (N=2903), n (%)
Ouagadougou	937 (32.28)
Lomé	467 (16.09)
Abidjan	411 (14.16)
Conakry	165 (5.68)
Niamey	139 (4.79)
Libreville	68 (2.34)
Bobo Dioulasso	62 (2.14)
Bamako	61 (2.1)
Cotonou	59 (2.03)
Kinshasa	44 (1.52)

**Table 4:** Illustrative findings from the Merci Mon Héros social media monitoring and program adjustments.

Social media monitoring finding	Program adjustment
Organic engagement yielded more engagement with the campaign than paid promotion alone.	Maintained paid promotional posts to direct traffic to the site Looked into free ways to pull people in (Instagram and Facebook stories, Tweetups with multiple organizations, song and poetry contest, and increasing responses to individual social media posts) Researched other organizations and individuals with whom to collaborate
Facebook fans were primarily male.	Shifted promotion campaigns toward young women Included increased calls for action targeting men, including messages around consent and talking to other men about reproductive health, etc.
Shorter videos increased view times.	Shortened video duration Created video screenshot teasers to increase the likelihood a video would be watched
Menstruation topical content and Facebook Lives have some of the highest engagement levels.	Decided to continue to include menstruation content and conduct at least 1 Facebook Live per month
The most negative sentiment tied to the campaign was associated with religious or cultural beliefs against premarital sex.	Included videos and Facebook Lives with religious leaders who promoted talking openly with young people about reproductive health Developed content about how social support to young parents is more beneficial than rejecting young (single) parents

## Discussion

The application of these 6 steps to inform the MMH campaign led to several challenges and lessons learned, reflecting the limitations of this methodology.

### Internet Users and Social Media Access

World Bank data indicate that internet penetration rates vary considerably in each of the 4 countries under review. Côte d’Ivoire’s internet penetration was estimated at approximately 36% in 2019. This compares to approximately 16%, 12%, and 5% in Burkina Faso (2017), Togo (2017), and Niger (2018), respectively [21]. Further, social media would be accessed by just a subset of the web-based population, with urban, socioeconomic, and education skews [3]. However, given that social media was one of the media chosen for campaign implementation, potential biases posed by the methodology used for this study do not differ to those posed by the web-based campaign itself.

### Topic Volumes

Due to the highly nuanced nature of the conversation, topic volumes should not be viewed as complete or exhaustive. First, the search strings were created to minimize irrelevant conversation in the analysis. However, it is unlikely that a search string will ever be completely exhaustive due to the vast combination of words that could be used to discuss the topic, especially across multiple languages.

Second, some posts could feasibly sit across multiple topics, yet they are assigned to just one to analyze guideline volumetrics for the conversation. However, given the same principles and classifications are applied consistently across the analysis, we view the results as representative of the total conversation.

### Topic Sources

The analysis was designed to extract the mentions of public-facing social media platforms, including Twitter, YouTube comments, forums, blogs, Reddit, Pinterest, and Tumblr, etc. Notable exemptions from this list are Facebook, Instagram, and WhatsApp. Social listening tools such as Crimson Hexagon cannot track these sites due to their privacy policies. The only Facebook page that was included in this analysis is the Merci Mon Héros campaign page. Giving social listening analysts administrative access to campaign pages is crucial to be able to effectively conduct social media monitoring and social listening on Facebook.

### Machine Learning

Processes that involve machine learning should not be considered “standardized” given that the algorithms used for these analyses are constantly learning. As such, the machine becomes more accurate over time as it continues to understand the nuance within the topic material.

### Lessons Learned

There are many important differences between traditional research methodologies and social media monitoring and listening, yet each adds useful elements to the monitoring and evaluation of health campaigns. Population-based quantitative surveys allow researchers to develop findings that are generalizable and standardized and enable data disaggregation. Traditional qualitative research techniques allow for in-depth probing to explore and understand the themes of interest. In contrast, social listening techniques enable users to rapidly synthesize the universe of web-based chatter around selected topics. Demographic data for individual posts are not accessible, making data interpretation more challenging. Techniques for identifying sex, age, and socioeconomic status are evolving, mostly based on analyzing the keywords and account activity associated with individual profiles. As artificial intelligence becomes more sophisticated, social listening platforms will improve at detecting the demographic detail of users, and thus, this technology’s use in research will continue to require careful ethical consideration.

Nonetheless, social media listening data can be quantified and tracked over time and used to retrospectively and prospectively analyze the data. Qualitative themes can be assessed, although these techniques do not allow for additional probing for clarification or the more nuanced understanding achieved by real-time traditional qualitative techniques. The following lessons were learned from the application of social media monitoring and social listening to the MMH campaign.

Future public health social media campaigns must:

- Understand who uses social media in the implementation countries and consider how the campaign’s target audience and content align with the audiences that are active on social media platforms.
- Assess which social media platforms are most active and relevant in the country of interest and the privacy limitations associated with these platforms. Relatedly, if Facebook or Instagram are the key platforms for the target audiences in the country, it is crucial that social listening analysts have administrative access to campaign pages.
- Pilot multiple engagement strategies adapted to the social media channel (ie, Facebook and Instagram, etc.) to test, through social monitoring and listening, which strategies are associated with higher engagement in adolescent sexual health–related posts.
- Pair designated hashtags representing the goals of the campaign to facilitate the monitoring of conversations. The implementation of these hashtags should be consistent across social media channels.

### Conclusion

Social listening and social media monitoring are effective monitoring and evaluation support tools that can be used to aid adaptive management. With the rise in internet and social media penetration as well as the accelerated development of artificial intelligence to enhance rapid data extraction and analysis tools, these methodologies will become increasingly relevant for public health research and evaluation. Researchers should continue to look for tools that minimize or eliminate the need for in-person data collection to avoid disruptions to data collection such as those experienced at the onset of the COVID-19 pandemic. Inherent biases that exist around internet and social media access are important challenges that limit these methodologies. Additionally, ethical considerations around data privacy must continue to guide advances in this technology’s use for research. However, for health communication campaigns that already engage populations who have a digital presence, social listening and social media monitoring can be powerful monitoring and evaluation tools.

## Abbreviations

FP: family planning
LMIC: low- and middle-income countries
MMH: Merci Mon Héros
RH: reproductive health
SRH: sexual and reproductive health
USAID: U.S. Agency for International Development
WABA: West Africa Breakthrough ACTION
